# Influence of levels of automation on the sense of agency during continuous action

**DOI:** 10.1038/s41598-021-82036-3

**Published:** 2021-01-28

**Authors:** Sayako Ueda, Ryoichi Nakashima, Takatsune Kumada

**Affiliations:** 1 TOYOTA Collaboration Center, RIKEN Center for Brain Science, Wako, Japan; 2 Graduate School of Informatics, Kyoto University, Kyoto, Japan

**Keywords:** Psychology, Human behaviour

## Abstract

Recent advances in automation technology can lead to unsafe situations where operators lose their sense of agency over the automated equipment. On the other hand, increasing evidence has shown that providing operators with opportunities of continuous operation and helping them improve their performance on tasks through automation can boost their sense of agency. However, it is challenging to ensure that the operator maintains a sense of agency when working with a fully automated tool that removes him/her from the control loop. By demonstrating a tracking task in which participants continuously tracked a moving target through a cursor controlled by a joystick under different levels of automation, we illustrate how the participants’ sense of agency and tracking performance were altered in accordance with the level of automation. The results showed that their sense of agency was enhanced by increasing automation but began to decline when the level of automation exceeded 90%. More generally, this suggests that allowing operators a little contribution to control over the continuous operation of an automated tool may be sufficient to maintain their sense of agency while yielding the maximum improvement in performance.

## Introduction

Recent advances in automation technology to reduce the workload on operators, operational costs, and errors have made everyday life easier and safer, but have also created novel and complicated problems. For example, automation technology seems to take human operators out of the control loop of systems by simply reducing their opportunities for direct control, which sometimes drastically impacts performance, especially in the case of system failure. The lessons of some accidents (for example, the Three Mile Island nuclear accident and the crash of Air France Flight 447 in 2009) suggest that operators may not be able to examine the situation, find appropriate solutions, and retake control when the automated equipment fails. Keeping operators in the control loop, even when their opportunity for direct control is reduced, is a central requirement for the safe design of applications of automation.

The operator’s loss of a sense of agency over the automated equipment has been proposed as one cause of the out-of-the-loop performance problem^1^. The sense of agency refers to the experience of controlling one’s own actions and, through them, events in the world^2,3^. It enables actions to be correctly identified as one’s “own” or “other”^4,5^, and is linked to feelings of responsibility and regret for actions and their outcomes^5,6^. In experimental studies, the sense of agency has commonly been quantified either by explicit ratings—asking participants to directly judge their own notion of control in a given task—or by implicit measures that use indirect indications of sense of agency in behavioral tasks^2,7–9^. In a pioneering study on the sense of agency over automated equipment, Berberian et al.^10^ used a flight simulator to show that the participants’ sense of agency when performing an aircraft navigation task decreased with increasing levels of automation. The task in their experiment was to avoid a collision with another plane by changing the aircraft’s trajectory. The participants identified the appropriate heading command when they detected the threat of collision (heading decision), implemented the command using a scroll wheel (heading implementation), and finally executed it by pressing an engagement button (command engagement). Feedback concerning the success of the action was then presented to them. The levels of automation were varied from complete control by the participant to full automation. In the complete control level, no procedure was automated. In the automatic decision level, the heading decision was automated. In the automatic decision and implementation level, the heading decision and the heading implementation were automated. Finally, in the full automation level, the entire procedure was fully automated while the participant simply observed it. The participants’ sense of agency was directly measured by their verbal reports on how strongly they felt that they had performed the operation to avoid collision. According to this, their sense of agency weakened as the procedure became more automated. Exploring ways to maintain the operator’s sense of agency over the automated equipment is thus important for designing successful applications that can keep operators in the control loop.

The combination of two measures can be taken to maintain the operators’ sense of agency over the automated equipment: To provide operators with opportunities for continuous operation of the equipment even if this is not entirely reflected in the output, and to help them improve their performance on tasks through automation. Some studies have shown that improving the performance of operators at tasks that involve the continuous control of a tool by using automatic assistance can help enhance, rather than diminish, their sense of agency^11,12^. Wen et al*.*^11^, for instance, showed that the sense of agency increases with an improvement in performance induced by unnoticed assistance by a computer. In their study, the participants were asked to place a dot within a goal (a square) on a computer screen as quickly as possible. They controlled the movement of the dot by continuously pressing the left and right keys on a keyboard. Two conditions were prepared: complete control by the participant, and computer-assisted control. In the complete control condition, all key presses were reflected in the movement of the dot. In the computer-assisted condition, the movement of the dot was modified so that it could not move away from the goal. The latter condition yielded better performance. After each trial, the participants were asked about the extent to which they had felt that the dot was under their control. The results showed that their sense of agency was reportedly higher in the case of computer-assisted control than in the complete control condition. Interestingly, this observation of the increase in the sense of agency was replicated even when the participants were given instructions about the assistance^12^. In addition, Nataraj et al.^13^, by using a virtual reality environment in which the participants controlled a virtual hand to perform reach-to-grasp movements, showed that the sense of agency decreased if modifications in the movement of the virtual hand due to the automation degraded task performance. These results suggest a positive relationship between apparent task performance as modified by automation and the sense of agency in situations where people continuously operate a tool featuring some automation. However, the level of automation was not manipulated gradually in these studies. Therefore, in the situation when the automation increases the task performance, the optimal level of automation that can help maintain the operators’ sense of agency over the automated equipment in situations where they continuously operate it is unclear. To clarify this, the relationship between apparent task performance when modified by automation and the operator’s sense of agency needs to be determined at varying levels of automation gradually from complete control by the operator to full automation.

In this study, we investigated the relationship between apparent task performance and the operator’s sense of agency over a tool, in a situation in which he/she continuously operates the tool with various levels of automation. To this end, we used a tracking task where the participants were asked to continuously track a moving target on a computer screen with a cursor controlled by a joystick at different levels of automation, varying from complete control by the participant to full automation. All operations of the joystick were reflected in the movements of the cursor in the complete control condition, whereas the cursor automatically and correctly followed the target independently of joystick operation in the full automation condition. From previous findings^11,12^, the enhancement in the sense of agency with apparent tracking performance as modified by automation was predictable. However, it was considered unlikely that the participants would have maintained their sense of agency in the full automation condition, even if they continuously operated the joystick, because they actually would have had nothing to do with the apparent cursor movement (i.e., they had been taken out of the control loop). Therefore, we hypothesized that when the level of automation continued to rise to the full automation condition, even if the participants’ sense of agency increased with apparent tracking performance to a certain degree, the enhancements in this due to automation would cease at some point, and their sense of agency would start declining. Identifying the turning point where the participants’ sense of agency decreased to lower than that in the complete control condition is another aim of this study. We performed two step-wise experiments in light of these aims. In Experiment 1, we replicated the findings of past experiments^11–13^, whereby the sense of agency increases with an increase in apparent task performance due to automation and decreases in the full automation condition. In Experiment 2, we identified the turning point where the participants’ sense of agency decreases to lower than that in the complete control condition.

## Methods

### Participants

Sixty adults (Experiment 1: 11 females, 19 males; Experiment 2: 17 females, 13 males) aged 18 to 31 years (Experiment 1: M = 21.6 years, SD = 2.4 years; Experiment 2: M = 22 years, SD = 3.7 years) participated in this study and received monetary compensation for their participation. The sample size was decided by a power analysis using G*Power^14^, which indicated that a minimum sample size of 29 participants was needed to detect a medium effect (f = 0.25) with 95% power, using a within-subjects repeated-measures design ANOVA with an alpha of 0.05. All participants had normal or corrected-to-normal vision. Fifty-three participants (Experiment 1: 27 participants; Experiment 2: 26 participants) were right-handed according to the Edinburgh Handedness Inventory^15^. They were all ascertained to have normal visuomotor functions as assessed by the grooved pegboard test (Lafayette Instruments, Lafayette, IN). Written informed consent was obtained from all of them in accordance with a protocol approved by the RIKEN Research Ethics Committee [Wako3 28–17(4)]. All experiments were performed according to the relevant guidelines.

### Apparatus, setup, and procedure

**Figure 1:**
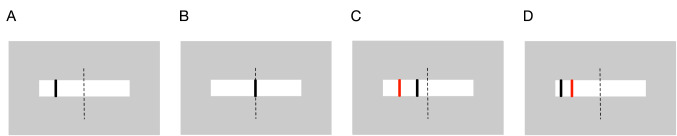
Task conditions. The target was a wide white bar and the cursor was a thin black bar. The target’s length varied over time and moved unpredictably in the horizontal direction. The participants were instructed to track the center of the target, represented as dotted lines, using the cursor. In the complete control condition (**A**), all joystick operations were reflected in the cursor movement. In the full automation condition (**B**), no joystick operation was reflected in the cursor movement, and the cursor was always at the center of the target. In the assistive automation conditions (**C**), the cursor was always displayed to reduce the tracking error, the distance between the center of the target and the position of the actual cursor, represented by the thin red bar. In the obstructive automation conditions (**D**), the cursor was always displayed to increase the tracking error (the actual cursor is again represented by the thin red bar). Participants could not see the actual cursor movement in this experiment.

The experiment was conducted in a dimly lit room. The participants sat 60 cm away from a 27-inch computer screen (2560 × 1440 pixels, 53.1° × 31.4° in terms of visual angle), with their heads fixed by a forehead-and-chin rest. The target and cursor were displayed on the computer screen. The target was a wide, white bar that moved horizontally (i.e., the x-coordinate was variable and the y-coordinate was fixed) on the computer screen. The target’s trajectory was generated as the sum of two sinusoidal waves in the horizontal plane so that participants could not predict it. In addition, the target’s width was varied from 541 to 2004 pixels over time as the sum of two sinusoidal functions with different frequencies independent of its movement. The cursor was a thin black bar (width × height: 5 × 50 pixels) located on the target (see Fig. 1A). The participants tracked the center of the target by controlling the cursor with a joystick (Logitech Extreme 3D Pro) using both hands. The angle of the joystick with respect to its original position corresponded linearly to the horizontal distance between the cursor and the center of the screen. The x-coordinate of the position of the cursor bar was recorded at intervals of 1/60 s. The presentation of the stimuli and the recording of the tracking trajectories of the participants were controlled by a computer with MATLAB software and its Psychtoolbox extension^16^.

The computer automatically modulated the movement of the cursor. The levels of automation were varied from complete control to full automation. In the complete control condition (Fig. 1A), all joystick operations were reflected in the cursor movement and, therefore, this condition was treated as the control condition. In the full automation condition (Fig. 1B), no joystick operation was reflected in the cursor movement, and the cursor automatically and correctly followed the center of the target (i.e., perfectly assisted control). For each experiment, we prepared four further conditions in terms of the levels of automation: the 33%, 66%, − 33%, and − 66% automation conditions for Experiment 1, and the 80%, 85%, 90%, and 95% automation conditions for Experiment 2. Each percentage represents the level of automation, which was defined as the degree of modulation by the computer. A higher percentage implies larger modulation. The positive sign represents assistive (i.e., where the computer helped the participants with tracking) automation (Fig. 1C) and the negative sign represents obstructive (i.e., the computer hindered the tracking by the participants) automation (Fig. 1D). For example, in the 33% automation condition, the tracking error (i.e., the distance between the center of the target and the cursor) always decreased by 33%, and led to an improvement of 33% in tracking performance over the complete control condition. However, in the − 33% automation condition, the tracking error always increased by 33%, and led to a decline of 33% in tracking performance compared with the complete control condition.

Each trial was initiated with the participant setting the joystick to the original position and pressing a switch on it. Each trial lasted 16.6 s and was divided into two epochs: a preparatory epoch (5 s) and a test epoch (11.6 s). In the preparatory epoch, the cursor was invisible, and the participants were asked to start tracking the center of the target without visual feedback from it. This phase was necessary so that the participants remained unaware of the initial, unnatural movement of the cursor induced by automation. In the full automation condition, for example, the cursor always started moving before the operation of the joystick. In the second test epoch, the cursor became visible and the participants could control it. The cursor became invisible again if a participant did not continue moving it for longer than one second to ensure its continuous operation throughout the trial. The participants had been informed of this setting before the experiment. After each trial, the participants rated the extent to which they had felt that the cursor was under their control (i.e., their sense of agency in terms of controlling it), and the extent to which they had felt that they performed well. The latter was intended to determine how they subjectively evaluated their apparent performance on the task as modified by automation; a seven-point scale was used (1 = not at all; 7 = a lot).

The participants were asked to accurately and smoothly track the center of the target by controlling the cursor during the trial. They were not notified about the automation of the cursor in the conditions. To familiarize them with the task settings and controlling the cursor using the joystick, the participants were asked to practice for 10 trials in the complete control condition before the experiment. Following the practice session, each participant completed 36 trials (experimental session), with six trials for each condition, in random order. Ten target trajectories were prepared for the practice session. For the experimental session, another six trajectories were prepared. The participants were allowed five-minute breaks between the practice and the experimental sessions. The experiment lasted for 20 min on average.

### Data analysis

The two self-reported ratings were separately submitted to one-way repeated measures analysis of variance (ANOVA) tests along with the condition used (i.e., for Experiment 1, the complete control condition, the full automation condition, and the − 33%, − 66%, 33%, and 66% automation conditions; for Experiment 2, the complete control condition, the full automation condition, and the 80%, 85%, 90%, and 95% automation conditions). When a significant effect for the given condition was noted, multiple subsequent comparisons were conducted using Shaffer’s modified version of the sequentially rejective Bonferroni procedure^17^. If a positive relationship was observed between the apparent task performance and the sense of agency, the control rating was predicted to increase with the degree of assistive automation but decrease with the degree of obstructive automation. If participants could not continue to maintain a sense of agency over the automated cursor, it is predicted that at some level of automation, the control rating would decrease to lower than that in the complete control condition. In addition, as follow-up analysis, we directly examined the relationship between the control ratings and the performance ratings. In this analysis, we calculated the individual Pearson’s correlation coefficients between the self-reported ratings and subjected them to a one-sample t-test to compare the mean individual correlation coefficients with the zero value. The significance threshold was set to *P* < 0.05 for all tests. Statistical analysis was conducted using R software (version 3.3.2. for Mac, R), and ANOVAs were executed using “anovakun” in R software (version 4.7.1.)^18^.

### Ethics approval and consent to participate

All experiments were conducted in accordance with a protocol approved by the RIKEN Research Ethics Committee (Wako3 28–17(4)).

## Results

### Experiment 1

**Figure 2:**
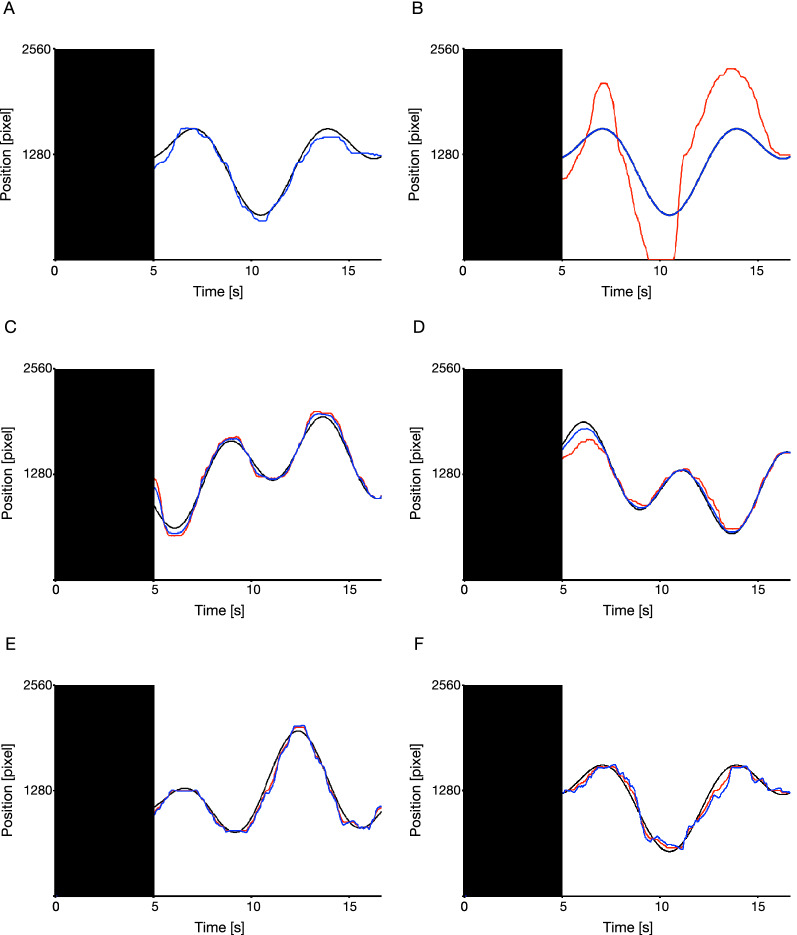
Examples of the movements of the target and the cursor in the complete control (**A**), full automation (**B**), 33% automation (**C**), 66% automation (**D**), − 33% automation (**E**), and − 66% automation conditions (**F**). Zero (2560) along the vertical axis indicates the left (right) side of the display. The black areas represent the preparatory epoch, where the cursor was invisible. The black curved lines represent the center of the target trajectories, and the colored lines represent the typical cursor movements of a participant (blue lines: the apparent cursor movements, red lines: the actual cursor movements). It is noted that participants could not see the actual cursor movement in this experiment.

Figure 2 illustrates examples of movements of the target and the apparent/actual cursor for each condition for a participant (complete control: Fig. 2A, full automation: Fig. 2B, 33% automation: Fig. 2C, 66% automation: Fig. 2D, − 33% automation: Fig. 2E, − 66% automation: Fig. 2F). The apparent cursor movements (the blue line) reflected modulation by the computer while the actual cursor movements (the red line) reflected all of the participant’s operations on the joystick. Therefore, only the apparent cursor movements were presented to the participants throughout the experiments (i.e., the apparent cursor was visible but the actual cursor was invisible). In Fig. 2A, the apparent cursor movements (the blue line) reflected all of the participant’s operations on the joystick because the apparent cursor movements were identical to the actual cursor movements in the complete control condition. The apparent cursor movements show how each automation condition modified the actual cursor movements. The actual cursor movements in the 33%, 66%, − 33%, and − 66% automation conditions appeared to follow the target trajectory comparably to that in the complete control condition, regardless of whether the automation was assistive or obstructive. However, in the full automation condition, the actual cursor movements seemed to clearly fail to follow the target trajectory.

**Figure 3:**
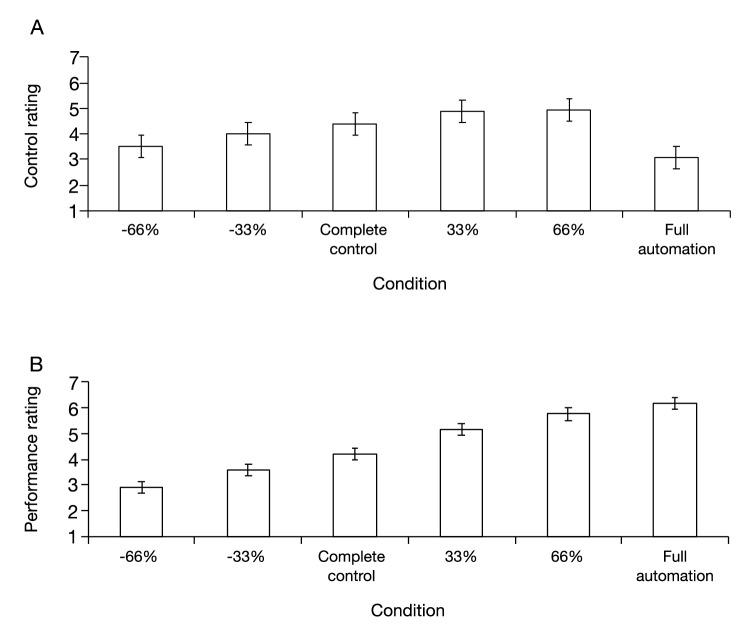
Average self-reported ratings for each condition. Statistical comparisons revealed that the full automation condition was significantly lower than the complete control condition in terms of control rating (**A**), but higher in terms of performance rating (**B**). The percentages − 66%, − 33%, “Complete control,” 33%, 66%, and “Full automation” denote the − 66% automation, − 33% automation, complete control, 33% automation, 66% automation, and full automation conditions, respectively. Error bars represent 95% within-subjects confidence intervals^39^.

Figure 3A,B show the average self-reported ratings of the participant for each condition. Except in the full automation condition, the control rating (Fig. 3A) and the performance rating (Fig. 3B) showed similar trends, i.e., they increased with the degree of assistive automation and decreased with the degree of obstructive automation. However, the increase approached a plateau after the 33% automation condition in the control rating but remained stable in the performance rating. In the full automation condition, the control rating suddenly decreased while the performance rating continued to rise. To assess these trends statistically, we performed one-way repeated ANOVA tests separately for each rating. They yielded significant effects of the task conditions on the control ratings [*F*(5, 145) = 11.70, *p* < 0.01, *ηp*^2^ = 0.29] and performance ratings [*F*(5, 145) = 118.67, *p* < 0.01, *ηp*^2^ = 0.80]. The results of all post-hoc tests are given in Table 1 for the control rating and in Table 2 for the performance rating. They reveal mostly expected trends. Specifically, compared with that in the complete control condition, the control rating was lower in the − 66% automation condition but not significantly different in the − 33% automation condition, higher in the 33% automation condition but not significantly different in the 66% automation condition, and lower in the full automation condition. Moreover, the control rating was lower in the − 66% condition than in the − 33% automation condition, but there was no significant difference between the 33% and 66% automation conditions. This suggests that the sense of agency increased with apparent tracking performance, but stopped increasing, or approached a plateau, after the 33% automation condition, and decreased, in the full automation condition, to lower than that in the complete control condition. In contrast, compared with that in the complete control condition, the performance rating was lower in the − 66% and − 33% automation conditions, and higher in the 33%, 66%, and full automation conditions. Moreover, the performance rating was lower in the − 66% condition than the − 33% automation condition, higher in the 66% condition than the 33% automation condition, and marginally higher in the full automation condition than the 66% automation condition. This suggests that the participants reported their apparent performance as modified by automation.

**Table 1:** Multiple comparison using Shaffer’s modified sequentially rejective Bonferroni procedure: *Control ratings.*

Pair			*T*	*Adjusted P*	d
**Experiment 1**					
− 66%	<	− 33%	3.13	0.03	0.57
− 66%	<	Complete	4.11	< 0.01	0.75
− 66%	<	33%	5.08	< 0.01	0.93
− 66%	<	66%	4.01	< 0.01	0.73
− 66%	n.s	Automation	0.96	0.69	0.17
− 33%	(<)	Complete	2.44	0.08	0.44
− 33%	<	33%	4.27	< 0.01	0.78
− 33%	<	66%	3.07	0.03	0.56
− 33%	n.s	Automation	2.09	0.16	0.38
Complete	<	33%	3.58	< 0.01	0.65
Complete	n.s	66%	2.16	0.16	0.39
Complete	>	Automation	3.19	0.02	0.58
33%	n.s	66%	0.48	0.69	0.09
33%	>	Automation	4.52	< 0.01	0.82
66%	>	Automation	5.39	< 0.01	0.98
**Experiment 2**					
Complete	n.s	80%	1.80	0.49	0.33
Complete	n.s	85%	1.73	0.49	0.32
Complete	n.s	90%	1.43	0.66	0.26
Complete	>	95%	2.96	0.04	0.54
Complete	>	Automation	3.52	0.01	0.64
80%	n.s	85%	0.42	1.00	0.08
80%	n.s	90%	0.07	1.00	0.01
80%	>	95%	4.22	< 0.01	0.77
80%	>	Automation	4.89	< 0.01	0.89
85%	n.s	90%	0.31	1.00	0.06
85%	>	95%	5.09	< 0.01	0.93
85%	>	Automation	5.07	< 0.01	0.92
90%	>	95%	4.07	< 0.01	0.74
90%	>	Automation	5.43	< 0.01	0.99
95%	>	Automation	3.52	0.01	0.64

**Table 2:** Multiple comparison using Shaffer’s modified sequentially rejective Bonferroni procedure: *Performance ratings.*

Pair			*T*	*adjusted P*	d
**Experiment 1**					
− 66%	<	− 33%	6.82	< 0.01	1.25
− 66%	<	Complete	10.39	< 0.01	1.90
− 66%	<	33%	18.36	< 0.01	3.35
− 66%	<	66%	22.05	< 0.01	4.03
− 66%	<	Automation	13.09	< 0.01	2.39
− 33%	<	Complete	5.24	< 0.01	0.96
− 33%	<	33%	12.66	< 0.01	2.31
− 33%	<	66%	14.86	< 0.01	2.71
− 33%	<	Automation	11.31	< 0.01	2.07
Complete	<	33%	9.39	< 0.01	1.71
Complete	<	66%	11.55	< 0.01	2.11
Complete	<	Automation	8.95	< 0.01	1.63
33%	<	66%	5.38	< 0.01	0.98
33%	<	Automation	4.38	< 0.01	0.80
66%	(<)	Automation	1.84	0.08	0.34
**Experiment 2**					
Complete	<	80%	11.22	< 0.01	2.05
Complete	<	85%	9.91	< 0.01	1.81
Complete	<	90%	11.06	< 0.01	2.02
Complete	<	95%	9.67	< 0.01	1.77
Complete	<	Automation	10.75	< 0.01	1.96
80%	<	85%	2.76	0.04	0.50
80%	<	90%	3.68	< 0.01	0.67
80%	<	95%	3.68	< 0.01	0.67
80%	<	Automation	6.13	< 0.01	1.12
85%	n.s	90%	1.19	0.26	0.22
85%	n.s	95%	2.25	0.10	0.41
85%	<	Automation	5.21	< 0.01	0.95
90%	n.s	95%	1.56	0.26	0.28
90%	<	Automation	4.22	< 0.01	0.77
95%	<	Automation	2.85	0.03	0.52

### Experiment 2

**Figure 4:**
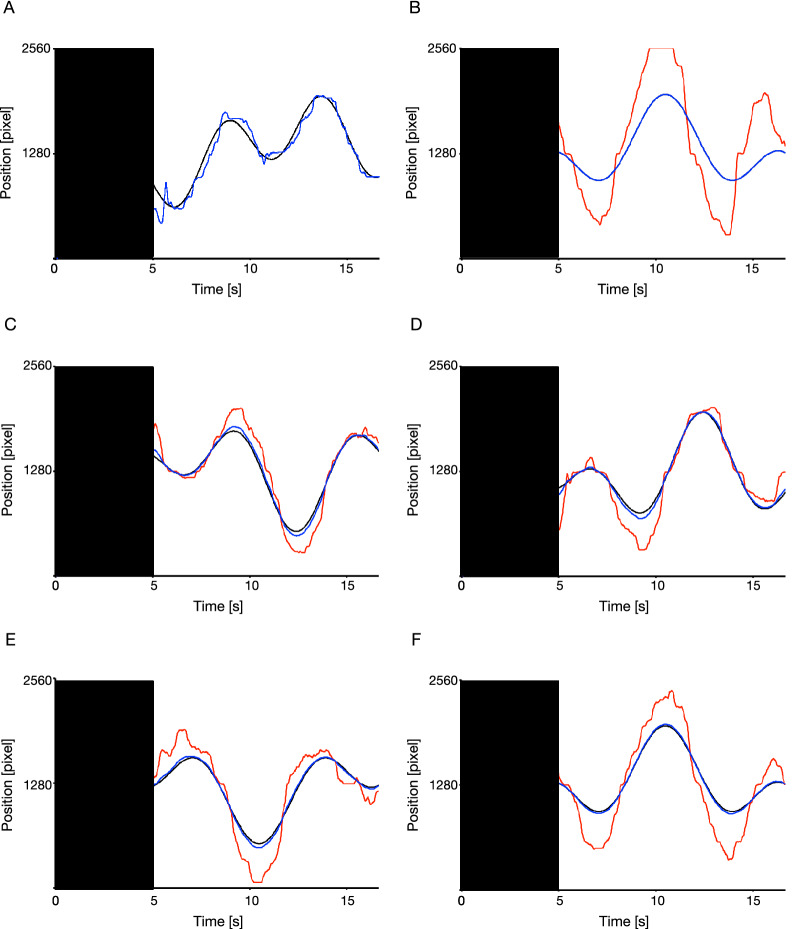
Examples of the movements of the target and cursor in the complete control (**A**), full automation (**B**), 80% automation (**C**), 85% automation (**D**), 90% automation (**E**), and 95% automation conditions (**F**). Zero (2560) along the vertical axis indicates the left (right) side of the display. The black areas represent the preparatory epoch where the cursor was invisible. The black curved lines show the center of the target trajectories and colored lines show the typical cursor movements of a participant (blue lines: the apparent cursor movements, red lines: the actual cursor movements). It is noted that participants could not see the actual cursor movement in this experiment.

Figure 4 illustrates examples of movement of the target and the apparent/actual cursor for each condition for a participant (complete control: Fig. 4A, full automation: Fig. 4B, 80% automation: Fig. 4C, 85% automation: Fig. 4D, 90% automation: Fig. 4E, 95% automation: Fig. 4F). The actual cursor movements increasingly deviated from the target trajectory as the extent of assistive automation increased.

**Figure 5:**
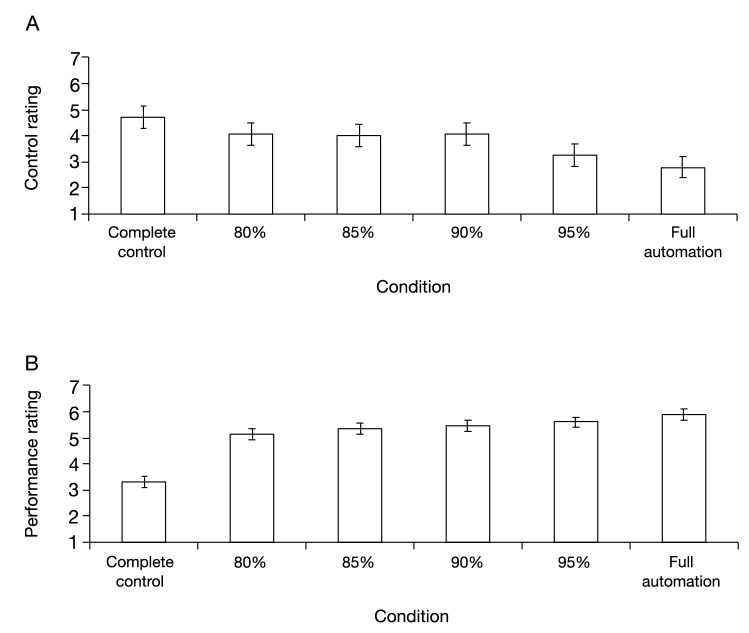
Average self-reported ratings for each condition. Statistical comparisons revealed that the 95% and the full automation conditions were significantly lower than the complete control condition in terms of control rating (**A**), but higher in terms of performance rating (**B**). “Complete control,” 80%, 85%, 90%, 95%, and “Full automation” denote the complete control, 80% automation, 85% automation, 90% automation, 95% automation, and full automation conditions, respectively. Error bars represent 95% within-subjects confidence intervals^39^.

Figure 5 shows the average self-reported ratings for each condition. The control rating (Fig. 5A) started to decrease gradually before the 80% automation condition and decreased further after the 90% automation condition. However, the performance rating (Fig. 5B) continued to gradually increase with the degree of assistive automation. To assess these trends statistically, we performed one-way repeated ANOVA tests separately for each rating. The results showed a significant effect of task condition on the control rating [*F*(5, 145) = 9.75, *p* < 0.01, *ηp*^2^ = 0.25] and performance [*F*(5, 145) = 78.32, *p* < 0.01, *ηp*^2^ = 0.72]. The results of all post-hoc tests are given in Table 1 for the control rating and Table 2 for the performance rating, each of which revealed the expected trends. Specifically, the control ratings in the 80%, 85%, and 90% automation conditions were not significantly different from those in the complete control condition, but lower than those in the 95% automation and full automation conditions. This suggests that the participants’ sense of agency decreased to lower than that in the complete control condition beyond the 90% automation condition. However, the performance rating was higher in the 85% automation condition than the 80% automation condition and complete control condition, and not significantly different from those in the 85%, 90%, and 95% automation conditions. The performance rating was also higher in the full automation condition than the 95% automation condition, suggesting that the participants reported their apparent performance as modified by automation.

### Follow-up analysis: correlation between the ratings

**Figure 6:**
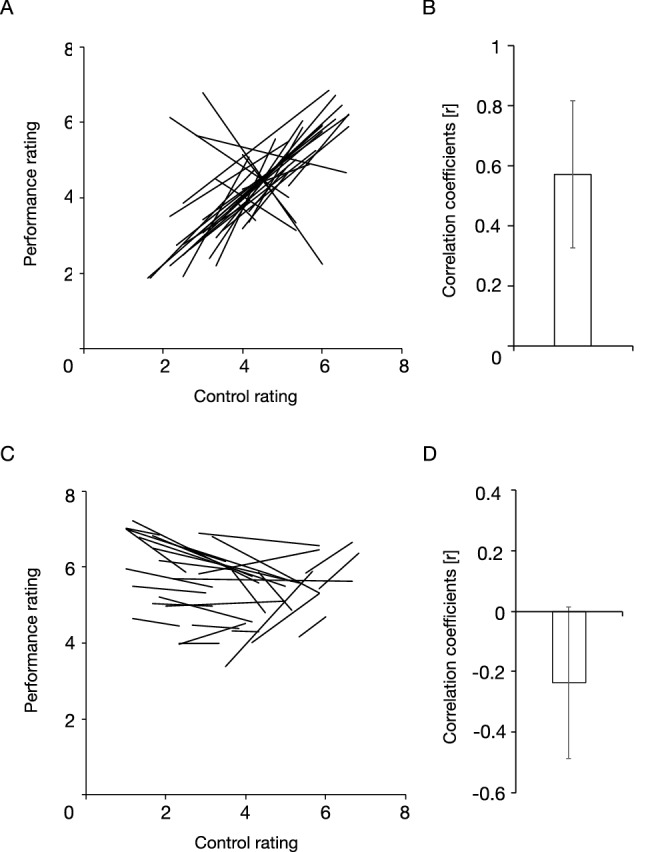
Regression lines fitted to each participant’s average self-reported ratings for the complete control condition and the − 33%, − 66%, 33%, and 66% automation conditions in Experiment 1 (**A**) and for the full automation condition, and the 80%, 85%, 90%, and 95% automation conditions in Experiment 2 (**C**). Average correlation coefficients between the control and the performance ratings for Experiment 1 (**B**) and Experiment 2 (**D**). The slopes of a majority of the regression lines seem to be positive in Experiment 1 (**A**) but weakly negative in Experiment 2 (**C**). The individual correlation coefficients between the self-reported ratings were significantly higher than the zero value for Experiment 1 (**B**) but marginally lower than zero for Experiment 2 (**D**). Error bars represent 95% confidence intervals.

The results of Experiments 1 and 2 showed that, as an overall trend, the relationship between the control and the performance ratings seemed to be positively correlated from the − 66% automation condition to the 66% automation condition but negatively correlated from the 80% automation condition to the full automation condition. Then, we examined whether these trends were observed within an individual participant. We fitted regression lines to each participant’s average self-reported ratings for each condition (i.e., for Experiment 1, the complete control condition and the − 33%, − 66%, 33%, and 66% automation conditions; for Experiment 2, the full automation condition, and the 80%, 85%, 90%, and 95% automation conditions): (the performance rating) = w0 + w1 × (the control rating). Figure 6A shows the regression lines for Experiment 1. The slopes of a majority of lines appeared positive. A one-sample t-test showed that the individual correlation coefficients between these ratings, shown in Fig. 6B, were significantly higher than zero [*t*(29) = 8.81, *p* < 0.01, *d* = 0.89]. This indicates a positive relationship between the subjective evaluations of apparent tracking performance and the sense of agency from the − 66% automation condition until the 66% automation condition. Figure 6C shows the regression lines for Experiment 2. The slopes of a majority of lines were weakly negative. The one-sample t-test showed that the individual correlation coefficients between these ratings, shown in Fig. 6D, were marginally lower than zero [*t*(29) =  − 1.94, *p* = 0.06, *d* =  − 0.34]. This indicates a weak negative relationship between subjective evaluation of apparent tracking performance and the sense of agency at levels of automation of 80% and higher.

To summarize, in Experiment 1, we replicated the findings of past research regarding the positive relationship between apparent task performance modified by automation and the sense of agency and confirmed the decrease of the sense of agency in the full automation condition. Furthermore, the increase in the sense of agency seemed to approach a plateau after the 33% automation condition. In Experiment 2, we observed a positive relationship between the apparent task performance as modified by automation and the sense of agency, which then became weakly negative at levels of automation of 80% and higher. The turning point, where the participants’ sense of agency significantly decreased to lower than that in the complete control condition, occurred beyond the 90% automation condition.

## Discussion

Recent developments in automation technology have made our lives easier but have also created unsafe situations where operators are taken out of the control loop and, therefore, lose their sense of agency over automated equipment. On the other hand, increasing evidence has shown that providing operators with opportunities of continuous operation and helping them improve their performance on tasks through automation can boost their sense of agency. However, it is challenging to ensure that the operator maintains a sense of agency when working with a fully automated tool that removes him/her from the control loop, even when the automated tool improves the task performance. There should be a turning point at which the sense of agency of the operator over the automated tool declines if the level of automation is increased gradually. No study to date has examined the relationship between apparent task performance as modified by automation and the sense of agency of the operator in such a gradual manner. Clarifying the relationship between apparent task performance when modified by automation and the sense of agency, and identifying the turning point, may help us design better automated tools that can keep operators in the control loop.

In this study, we conducted a tracking task, in which the participants continuously tracked a moving target on a computer screen with a cursor controlled by a joystick under different levels of automation, from complete control to full automation. We examined how the participants’ sense of agency changed with apparent tracking performance as modified by automation. We noted a positive relationship between them until at least the 66% automation condition, a robust replication of previous findings^11–13,19^. This suggests that the sense of agency can be increased to some extent depending on the apparent task performance, even if the task is not performed entirely by the operator. However, and more importantly, this positive relationship turned into a weakly negative one at levels of automation of 80% and higher. The turning point, at which the sense of agency started to decline, was beyond the 90% automation condition.

The decline in the sense of agency with increasing automation at levels of high automation suggests that as the level of automation increased, the participants became unable to attribute the improvement in their tracking performance to their own operation. This phenomenon can be explained by the seminal theoretical framework of the generation of the sense of agency: the internal comparator model^20,21^. This model is supported by a substantial number of studies that have used a variety of paradigms and indices^22–28^. According to the model, a sense of agency is produced via a comparison between the predicted outcome of an action, generated by means of an internal forward model^29,30^, and its actual outcome. If the predicted and the actual outcomes match, people feel that the perceived event occurred through their actions, that is, they experience a sense of agency. Conversely, if there is a mismatch between the predicted and the actual outcomes, people experience a lower sense of agency. In this study, the participants predicted their actual tracking performance as the outcome of their operations on the joystick, and then compared it with their apparent tracking performance to evaluate their sense of agency over the (automated) cursor. If the error between these two measures was small, the participants could attribute the apparent tracking performance to their own operation and could feel that they had control over the automated cursor. However, if the error increased, they became unwilling to attribute their apparent tracking performance to their own operation, and their sense of agency declined. In this case, we hypothesize that the larger the error between the predicted and the apparent tracking performance, the lower the sense of agency experienced by the participants over the cursor. Of course, we cannot assess this hypothesis directly because the predicted tracking performance was not clear in the experimental setup. We assume actual tracking performance can represent an approximation of the predicted tracking performance because the participants could have predicted the position of the invisible cursor on the basis of an internal forward model of the relationship between the cursor and the operation of the joystick, which might have been acquired during the practice session. Figure 4 shows that the movements of the actual cursor deviated increasingly from the target trajectory as the extent of assistive automation increased. We therefore analyzed post hoc the relationship between the error between the actual and the apparent tracking performance (i.e., the performance error) and the sense of agency using the data from Experiment 2 (see the Supplementary Information 1 for details). The results showed a negative relationship between performance error and the sense of agency, suggesting that as the error between the predicted and the apparent tracking performance increased, the participants became unwilling to attribute the apparent tracking performance to their operation, and felt a lower sense of agency. However, this suggestion should be confirmed directly in future studies.

The findings of the present study suggest that the sense of agency can be modulated by both internal comparison and task performance. This is in agreement with recent models of the generation of the sense of agency^9,31^ that propose an interactive relationship between the process of internal comparison and the higher-level cognitive process concerning external cues, such as an improvement in task performance and knowledge of automation, to generate a sense of agency. In particular, the cue-integration theory^31^ proposes that the extent to which these two processes contribute to the sense of agency is determined by their reliability. This model can be used to show that the turning point, where participants began to lose their sense of agency over the automated cursor, occurred due to a change in balance between the reliability of the processes of internal comparison and higher-level cognition. That is, when the level of automation was low, as in the 33% automation condition, information on the internal comparison (i.e., the error between the predicted and apparent performance) was ambiguous because the actual cursor was invisible to the participants. Therefore, the process of internal comparison was judged to have been unreliable and to have contributed less to the sense of agency than the higher-level cognitive process. However, once the level of automation increased to the 95% condition, error-related information increased significantly, and the internal comparison process was considered sufficiently reliable to predominantly contribute to the sense of agency.

An interesting concern regarding the application of our findings is related to the generality of the turning point. In this study, the participants’ sense of agency began declining beyond the 90% automation condition, suggesting allowing operators a little contribution to control over automated equipment during continuous operation is sufficient to maintain their sense of agency over it. This can help design safe and efficient applications for human–computer interaction, which may prevent the out-of-the-loop performance problem^1^ and the loss of mental engagement of operators^32,33^. However, whether this level of automation can be generalized to other situations, where different automated tools are used or different tasks are performed, remains unknown. If the turning point is determined by a change in balance between the reliability of the processes of internal comparison and higher-level cognition, it should be changeable. For example, operators may be able to maintain their sense of agency until a level of automation higher than 90% when using automated equipment where the outcome of the operation is not easily predictable, such as a tanker ship with a long operational time delay, because the internal error must be ambiguous in such a situation and, therefore, the internal comparison process must be unreliable. This can be checked by examining the change in the turning point as associated with the change in the predictability of the outcome of an automated tool. Another concern regarding the application of our findings is the possibility that the use of an automated tool might change the previously acquired skill used for it. It is well known that people are capable of adaptively updating their internal models for tools^34–36^. In the case of using a tool with automation, it is not clear whether such use overwrites the previously acquired internal model. This impact of automation should also be investigated for designing successful automated tools.

Finally, it is worth noting that there is an alternative explanation to the process of internal comparison because we have employed explicit rating where participants were asked directly judge their own notion of control to measure the sense of agency: the postdictive account of the process of internal comparison. According to an influential postdiction framework^37,38^, the sense of agency can be seen as the product of a fallible postdictive inference based on contextual information during and after the action. In this study, to evaluate the sense of agency over the cursor after the tracking has completed, participants could compare their apparent tracking performance with their actual tracking performance, where the latter was both predicted beforehand (as the outcome of their operations on the joystick) and postdictively inferred (based on their previous intentions or operations already performed). In this line, the presented results of modulating the sense of agency would also be explained quite well by both the postdictive account of the process of internal comparison and task performance. That is, the turning point would be determined by a change in balance between the reliability of the postdictive account of the processes of internal comparison and higher-level cognition. Further studies with more sophisticated experimental designs and methods are required to clarify this possibility.

## Conclusions

We have shown here that the operators’ sense of agency over an automated tool can be enhanced in the combination of following two ways. One is to provide operators with opportunities for continuous operation even if their actions are not entirely reflected in the outcome. The other is to help them improve their performance on tasks through automation. More importantly, their sense of agency was boosted by increasing automation but began to decline when the level of automation exceeded 90%. This suggests, more generally, that allowing operators a little contribution to control over an automated tool during its continuous operation may be sufficient to maintain their sense of agency while providing the maximum improvement in performance. Further work is required to establish the generalizability of the findings to practical settings.

## Supplementary Information

Supplementary Information 1.

Supplementary Information 2.

## Data Availability

The data that support the findings of this study are included in the Supplementary Information 2.

## References

[1] Endsley MR, Kiris EO (1995). The out-of-the-loop performance problem and level of control in automation. Hum. Factors.

[2] Haggard P (2017). Sense of agency in the human brain. Nat. Rev. Neurosci..

[3] Haggard P, Chambon V (2012). Sense of agency. Curr. Biol..

[4] Haggard P, Tsakiris M (2009). The experience of agency: feelings, judgments, and responsibility. Curr. Dir. Psychol. Sci..

[5] Moore JW (2016). What is the sense of agency and why does it matter?. Front. Psychol..

[6] Frith CD (2014). Action, agency and responsibility. Neuropsychologia.

[7] Schwarz KA, Burger S, Dignath D, Kunde W, Pfister R (2018). Action-effect binding and agency. Conscious Cogn..

[8] Schwarz KA, Weller L, Klaffehn AL, Pfister R (2019). The effects of action choice on temporal binding, agency ratings, and their correlation. Conscious Cogn..

[9] Synofzik M, Vosgerau G, Newen A (2008). Beyond the comparator model: a multifactorial two-step account of agency. Conscious Cogn..

[10] Berberian B, Sarrazin JC, Le Blaye P, Haggard P (2012). Automation technology and sense of control: a window on human agency. PLoS ONE.

[11] Wen W, Yamashita A, Asama H (2015). The sense of agency during continuous action: performance is more important than action-feedback association. PLoS ONE.

[12] Inoue K, Takeda Y, Kimura M (2017). Sense of agency in continuous action: assistance-induced performance improvement is self-attributed even with knowledge of assistance. Conscious Cogn..

[13] Nataraj R, Sanford S, Shah A, Liu M (2020). Agency and performance of reach-to-grasp with modified control of a virtual hand: implications for rehabilitation. Front. Hum. Neurosci..

[14] Faul F, Erdfelder E, Lang AG, Buchner A (2007). G* Power 3: A flexible statistical power analysis program for the social, behavioral, and biomedical sciences. Behav. Res. Methods.

[15] Oldfield RC (1971). The assessment and analysis of handedness: the Edinburgh inventory. Neuropsychologia.

[16] Brainard DH (1997). The psychophysics toolbox. Spat. Vis..

[17] Shaffer JP (1986). Modified sequentially rejective multiple test procedures. J. Am. Stat. Assoc..

[18] Anovakun (version 4.7.1), http://riseki.php.xdomain.jp/index.php/ (2015).

[19] Metcalfe J, Greene MJ (2007). Metacognition of agency. J. Exp. Psychol. Gen..

[20] Blakemore S-J, Frith CD, Wolpert DM (1999). Spatio-temporal prediction modulates the perception of self-produced stimuli. J. Cogn. Neurosci..

[21] Frith C, Blakemore S-J, Wolpert DM (2000). Explaining the symptoms of schizophrenia: abnormalities in the awareness of action. Brain Res. Rev..

[22] Ebert JP, Wegner DM (2010). Time warp: authorship shapes the perceived timing of actions and events. Conscious Cogn..

[23] Farrer C, Valentin G, Hupé JM (2013). The time windows of the sense of agency. Conscious Cogn..

[24] Hon N, Poh J-H, Soon C-S (2013). Preoccupied minds feel less control: Sense of agency is modulated by cognitive load. Conscious Cogn..

[25] Kawabe T (2013). Inferring sense of agency from the quantitative aspect of action outcome. Conscious Cogn..

[26] Kühn S, Nenchev I, Haggard P, Brass M, Gallinat J, Voss M (2011). Whodunnit? Electrophysiological correlates of agency judgements. PLoS ONE.

[27] Sato A, Yasuda A (2005). Illusion of sense of self-agency: discrepancy between the predicted and actual sensory consequences of actions modulates the sense of self-agency, but not the sense of self-ownership. Cognition.

[28] Wen W, Yamashita A, Asama H (2015). The influence of action-outcome delay and arousal on sense of agency and the intentional binding effect. Conscious Cogn..

[29] Wolpert DM, Ghahramani Z, Flanagan JR (2001). Perspectives and problems in motor learning. Trends Cogn. Sci..

[30] Wolpert DM, Ghahramani Z, Jordan MI (1995). An internal model for sensorimotor integration. Science.

[31] Moore JW, Fletcher PC (2012). Sense of agency in health and disease: a review of cue integration approaches. Conscious Cogn..

[32] Shastri, D., Fujiki, Y., Buffington, R., Tsiamyrtzis, P., & Pavlidis, I. O job can you return my mojo: improving human engagement and enjoyment in routine activities. *Proceedings of the SIGCHI Conference on Human Factors in Computing Systems* 2491–2498 (2010)

[33] Dcosta, M., Shastri, D., Tsiamyrtzis, P., & Pavlidis, I. Turning security monitoring into an engaging high performance task. *2016 IEEE Symposium on Technologies for Homeland Security (HST)* 1–2 (2016)

[34] Imamizu H, Miyauchi S, Tamada T, Sasaki Y, Takino R, Pütz B, Yoshioka T, Kawato M (2000). Human cerebellar activity reflecting an acquired internal model of a new tool. Nature.

[35] Kawato M (1999). Internal models for motor control and trajectory planning. Curr. Opin. Neurobiol..

[36] Wolpert DM, Kawato M (1998). Multiple paired forward and inverse models for motor control. Neural Netw..

[37] Wegner D (2002). The Illusion of Conscious Will.

[38] Wegner D (2003). The mind's best trick: how we experience conscious will. Trends Cogn. Sci..

[39] Loftus GR, Masson ME (1994). Using confidence intervals in within-subject designs. Psychon. Bull. Rev..

