# Supplementary material for: Influence of levels of automation on the sense of agency during continuous action
Source: Sci Rep. 2021 Jan 28;11:2436. doi: 10.1038/s41598-021-82036-3 (PMC7843606; doi:10.1038/s41598-021-82036-3)

**Supplementary information**

**Title**

Influence of Levels of Automation on the Sense of Agency during Continuous Action

**Authors**

Sayako Ueda^*1^, Ryoichi Nakashima ^1,2^, and Takatsune Kumada^1,2^

* Corresponding author.

**Author details**

^1^TOYOTA Collaboration Center, RIKEN Center for Brain Science, Wako, Japan.

^2^ Graduate School of Informatics, Kyoto University, Kyoto, Japan.

***Correspondence:**

**E-mail**: ueda385@brain.riken.jp

**Tel**: +81-48-462-1111 ext.7197

**Address**: RIKEN CBS-TOYOTA Collaboration Center, RIKEN, 2-1 Hirosawa, Wako-shi, Saitama, 351-0198, Japan.

**Supplementary data analysis**

For each trial, the error between the actual and the apparent tracking performance (i.e., the performance error) was evaluated using the error between the actual and the apparent tracking errors of the cursor: (the actual tracking error) – (the apparent tracking error). Each metric of the tracking error was defined as the root mean-square (of position) error (RMSE), the distance between the centers of the target and each cursor (i.e., the actual or the apparent cursor). This is a commonly used performance-related index for visuomotor tracking tasks used in previous studies^S1–3^. A larger tracking error implies less accurate tracking. Therefore, a larger performance error implies less accurate tracking in the actual than the apparent cursor.

The performance error was subjected to one-way repeated measures analysis of variance (ANOVA) tests for all conditions (i.e., the complete control condition, the full automation condition, and the 80%, 85%, 90%, and 95% automation conditions). When a significant effect of a condition was observed, multiple subsequent comparisons were performed by using Shaffer’s modification of the sequentially rejective Bonferroni procedure^17^. In the case of a negative relationship between the performance error and the sense of agency, the error was predicted to increase with the degree of assistive automation. In addition, to directly examine the relationship between the performance error and the control rating, we calculated the individual Spearman’s correlation coefficients between them, and conducted a one-sample t test to compare the means of the individual correlation coefficients with the zero value. The significance threshold was set to P < .05 for all tests. Statistical analysis was conducted using R software (version 3.3.2. for Mac, R), and ANOVAs were executed using “anovakun” in R software (version 4.7.1.)^18^.

**Supplementary results**

Supplementary Fig. S1A shows the average performance error for each condition. It appears to continue to increase with the degree of assistive automation. To assess this trend statistically, we performed a one-way repeated ANOVA test. The results showed a significant main effect for the task condition [*F*(5, 145) = 76.73, *p* < 0.01, *ηp*^2^ = 0.73]. The results of all subsequent post-hoc tests are given in Supplementary Table S1. They revealed the expected trend. Specifically, the performance error was higher in the case of 80% automation than in the complete control condition, in the 85% automation condition than the 80% condition, in the 90% automation condition than the 85% condition, in the 95% automation condition than the 90% condition, and was higher in the full automation condition than the 95% automation condition. This suggests that the performance error increased with the level of assistive automation.

Supplementary Figure S1B shows the regression lines fitted to each participant’s average performance error and control rating for each condition. We excluded the complete control condition from this fitting and the following calculations because the performance error was always zero in the complete control condition regardless of the participant. The slopes of a majority of lines appeared negative. A one-sample t-test showed that the individual correlation coefficients between the performance error and the control ratings, shown in Supplementary Figure S1C, were significantly lower than the zero value [*t*(29) = −5.41, *p* < 0.01, *d* = −0.98]. This indicates that a negative relationship obtained between the error and the sense of agency at levels of automation of 80% and higher.

To summarize, Experiment 2 showed that a negative relationship exists between the performance error and the sense of agency.

**Supplementary references**

1. Hill, H., & Raab, M. Analyzing a complex visuomotor tracking task with brain-electrical event related potentials. *Hum. Mov. Sci.* **24**(1), 1–30 (2005).
2. Raab, M., de Oliveira, R. F., Schorer, J., & Hegele, M. Adaptation of motor control strategies to environmental cues in a pursuit-tracking task. *Exp. Brain Res.* **228**(2), 155–160 (2013).
3. Ueda, S., Sakai, H., Ueno, K., Cheng, K. & Kumada, T. Cerebellar activation associated with model-based estimation of tool-use consequences. *Behav. Brain. Funct.* **15**(1), 8; 10.1186/s12993-019-0158-y (2019).

**
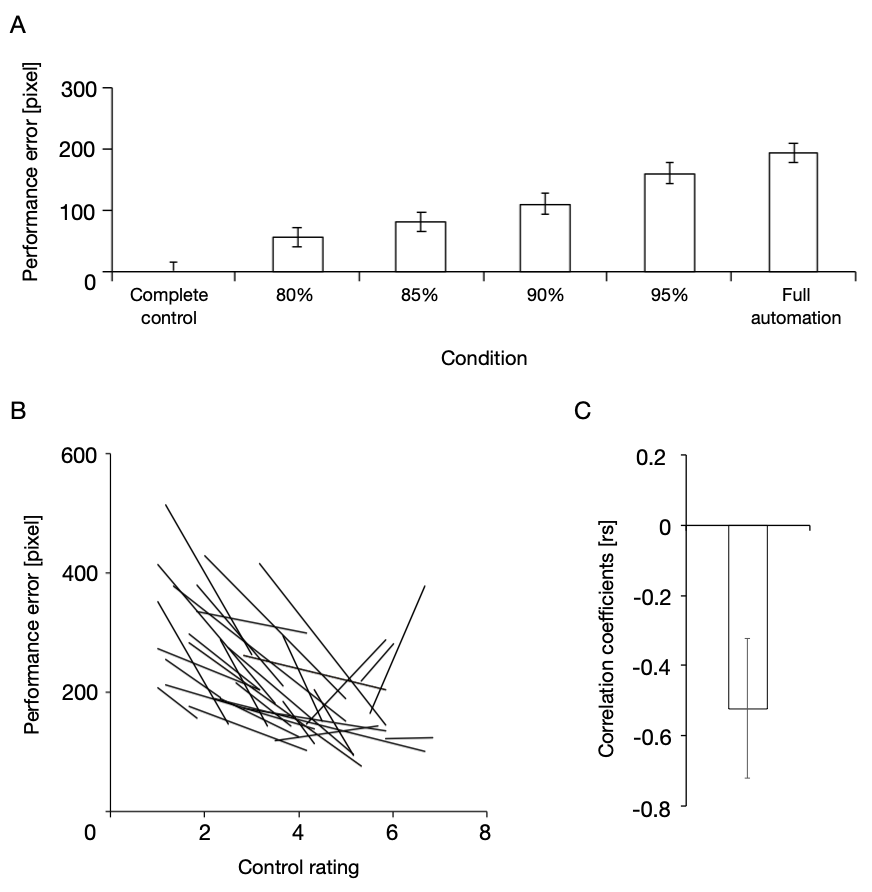
**

**Supplementary Figure S1**. Average performance error for each condition (A). Regression lines fitted to each participant’s average control rating and performance error for each condition except the complete control condition (B). Average individual correlation coefficients between the control rating and the performance error for all conditions except the complete control condition (C). Statistical comparisons revealed that the performance error increased with the level of assistive automation (A). The slopes of a majority of the regression seem to be negative (B). The individual correlation coefficients between the control rating and the performance error were marginally lower than zero (C). “Complete control,” 80%, 85%, 90%, 95%, and “Full automation” denote the complete control, 80% automation, 85% automation, 90% automation, 95% automation, and full automation conditions, respectively. Error bars represent 95% within-subjects confidence intervals^39^.


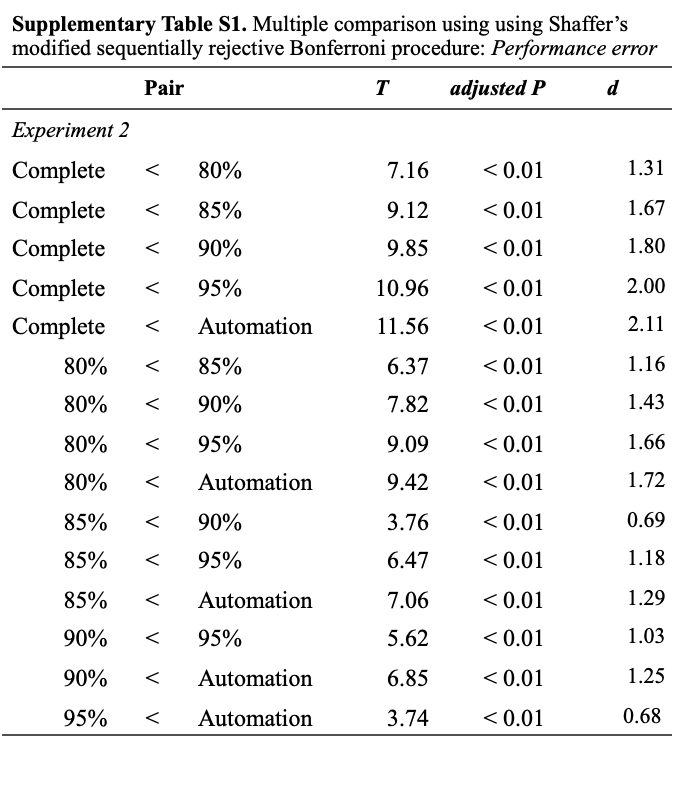

Supplement: Supplementary file 1 — Supplementary Information 1. [file 41598_2021_82036_MOESM1_ESM.docx]
